# A New Adaptive Self-Tuning Fourier Coefficients Algorithm for Periodic Torque Ripple Minimization in Permanent Magnet Synchronous Motors (PMSM)

**DOI:** 10.3390/s130303831

**Published:** 2013-03-19

**Authors:** Alfonso Gómez-Espinosa, Víctor M. Hernández-Guzmán, Manuel Bandala-Sánchez, Hugo Jiménez-Hernández, Edgar A. Rivas-Araiza, Juvenal Rodríguez-Reséndiz, Gilberto Herrera-Ruíz

**Affiliations:** 1 Centro de Ingeniería y Desarrollo Industrial, Dirección de Investigación y Posgrado. Av. Playa Pie de la Cuesta No. 702, Desarrollo San Pablo, C.P. 76130 Santiago de Querétaro, Qro., Mexico; E-Mails: mbandala@cidesi.mx (M.B.-S.); hugo.jimenez@cidesi.mx (H.J.-H.); 2 Universidad Autónoma de Querétaro, División de Estudios de Posgrado, Facultad de Ingeniería, Cerro de las Campanas s/n, C.P. 76010, Santiago de Querétaro, Qro., Mexico; E-Mails: vmhg@uaq.mx (V.M.H.-G.); erivas@uaq.mx (E.A.R.-A.); juvenal@ieee.org (J.R.-R.); gherrera@uaq.mx (G.H.-R.)

**Keywords:** torque ripple, frequency domain, FOC, self-tuning algorithm, PMSM, DSP

## Abstract

Torque ripple occurs in Permanent Magnet Synchronous Motors (PMSMs) due to the non-sinusoidal flux density distribution around the air-gap and variable magnetic reluctance of the air-gap due to the stator slots distribution. These torque ripples change periodically with rotor position and are apparent as speed variations, which degrade the PMSM drive performance, particularly at low speeds, because of low inertial filtering. In this paper, a new self-tuning algorithm is developed for determining the Fourier Series Controller coefficients with the aim of reducing the torque ripple in a PMSM, thus allowing for a smoother operation. This algorithm adjusts the controller parameters based on the component's harmonic distortion in time domain of the compensation signal. Experimental evaluation is performed on a DSP-controlled PMSM evaluation platform. Test results obtained validate the effectiveness of the proposed self-tuning algorithm, with the Fourier series expansion scheme, in reducing the torque ripple.

## 1. Introduction

The Permanent Magnet Synchronous Motor (PMSM) market is growing more rapidly when compared to traditional competitors because of lower cost, as well as higher efficiency and reliability. For the sake of energy savings and environmental performance, PMSMs also feature one of the highest torque to loss ratios. These motors are widely used in fast dynamic positioning systems and machine-tool components [1,2]. The main disadvantage of PMSMs is the non-uniformity in the developed torque, known as “torque ripple” [3]. Torque ripple generates speed oscillations which cause system performance deterioration and in machine-tool applications, it can leave visible patterns on high precision machined surfaces [4]. Under the assumption of pure sinusoidal back electromagnetic force (EMF), the conventional Field Oriented Control (FOC) applies constant current references in the synchronous reference frame to produce a constant torque. However, depending on the magnet shape and how well the windings are manufactured, the back-EMF has in practice very different waveforms, which range from almost sinusoidal to trapezoidal. Torque ripple occurs in PMSMs due to non-sinusoidal flux density distribution around the air-gap and the variable magnetic reluctance of the air-gap due to stator slots distribution. These torque ripples change periodically with rotor position and are apparent as speed variations, which degrades the PMSM drive performance, particularly at low speeds because of low inertial filtering [5].

In order to improve the performance of PMSMs and increase its market share, the suppression of the pulsating torque has received much attention in recent years [6–17]. These torque ripple reduction techniques can be divided into two groups: one focusing on the improvement of motor design and the other emphasizing the use of active control of stator current excitation. From the motor design point of view, skewing the stator lamination stacks or rotor magnets, arranging proper winding distributions and incorporating other motor design features reduces cogging torque partially, but does not completely eliminate it. Moreover, special machine design processes add addition complexity to the production process, which results in higher machine cost [18].

The second approach, which is of our interest, concentrates on using an additional control effort to compensate for the periodic torque pulsations. Some methods rely on pre-programed stator current excitation to cancel torque harmonics. However, accurate information about the PMSM parameters is required, and a small error or variation in these parameters can produce higher torque ripple due to the open-loop control. As an alternative, closed-loop control algorithms with online estimation of parameters and adaptive control algorithms have been proposed to reduce torque ripple. One possible approach relies on a closed-loop speed regulator to attenuate indirectly torque pulsations since all possible sources of torque ripple are observable from rotor speed, and hence this method has potential for complete torque ripple minimization. Repetitive Control techniques incorporate a sinusoidal control component to deal with periodic torque pulsations [19–23] while Iterative Learning Control (ILC) is implemented in the frequency domain to reduce torque ripple, by means of Fourier series expansion [24–29]. Some recent papers deal with learning control algorithms for Permanent Magnet Step Motors [30–32], by identifying the Fourier coefficients of any truncated approximation and implementing Iterative Learning techniques, providing an experimental comparison for both methods. Adaptive techniques have been proposed based on the spectrum of the torque perturbation using some theoretical developments.

In this paper, a new self-tuning algorithm is developed for determining the Fourier Series Controller coefficients with the aim of reducing the torque ripple in a PMSM, thus allowing for a smoother operation. This algorithm adjusts the controller parameters based on the component's harmonic distortion in the time domain of the compensation signal. The estimated Fourier coefficients are used by a nonlinear controller which achieves accurate and ripple-reduced torque control. Experimental evaluation was performed on a DSP-controlled PMSM evaluation platform and test results obtained verify the effectiveness of proposed self-tuning algorithm, with the Fourier series expansion scheme, in reducing the torque ripple.

This paper is organized as follows: A model of the Permanent Magnet Synchronous Motor is presented in Section 2. The new self-tuning Fourier Coefficient Algorithm is introduced in Section 3. Section 4 describes the experimental setup, and the experimental results are presented in Section 5. Finally, in Section 6 concluding remarks are provided.

## 2. Model of PMSM

In this section, a standard PMSM model [33] is revised and additional considerations are explained so torque ripple sources are clarified. For a three-phase PMSM, the flux linkages Ψ*_abs_* related to the mutual and self-inductances *Ls* and currents *i* are given as:

(1) $$\Psi_{\text{abcs}}=L_{s}i_{\text{abcs}}+\Psi_{m}$$

In matrix form:

(2) $$\begin{array}{c} \Psi_{\text{as}}=L_{\text{asas}}i_{\text{as}}+L_{\text{asbs}}i_{\text{bs}}+L_{\text{ascs}}i_{\text{cs}}+\Psi_{\text{asm}}\\ \Psi_{\text{bs}}=L_{\text{bsas}}i_{\text{as}}+L_{\text{bsbs}}i_{\text{bs}}+L_{\text{bscs}}i_{\text{cs}}+\Psi_{\text{bsm}}\\ \Psi_{\text{cs}}=L_{\text{csas}}i_{\text{as}}+L_{\text{csbs}}i_{\text{bs}}+L_{\text{cscs}}i_{\text{cs}}+\Psi_{\text{csm}} \end{array}$$

The stator windings voltages **u***_abcs_* depend on the winding resistance ***r****_s_* and flux linkages **Ψ***_abcs_*:

(3) $$u_{\text{abcs}}=r_{s}i_{\text{abcs}}+\frac{d\Psi_{\text{abcs}}}{dt}$$

Rewriting this expression in matrix form:

(4) $$\left[\begin{array}{c} u_{\text{as}}\\ u_{\text{bs}}\\ u_{\text{cs}} \end{array}\right]=\left[\begin{array}{lll} r_{s} & 0 & 0\\ 0 & r_{s} & 0\\ 0 & 0 & r_{s} \end{array}\right]\;\left[\begin{array}{c} i_{\text{as}}\\ i_{\text{bs}}\\ i_{\text{cs}} \end{array}\right]+\left[\begin{array}{c} \frac{d\Psi_{\text{as}}}{dt}\\ \frac{d\Psi_{\text{bs}}}{dt}\\ \frac{d\Psi_{\text{cs}}}{dt} \end{array}\right]$$

The stator windings are displaced by 120°, and the flux linkages Ψ*_asm_*, Ψ*_bsm_*, Ψ*_csm_* established by the permanent magnet, which are periodic functions of *θ_r_*, are assumed to be sinusoidal with magnitude Ψ*_m_*:

(5) $$\begin{array}{c} \Psi_{\text{asm}}=\Psi_{m}\sin(\theta_{r})\\ \Psi_{\text{bsm}}=\Psi_{m}\sin\left(\theta_{r}-\frac{2}{3}\pi \right)\\ \Psi_{\text{csm}}=\Psi_{m}\sin\left(\theta_{r}-\frac{2}{3}\pi \right) \end{array}$$

From Equation (3), assuming ***L****_s_* is constant:

(6) $$u_{\text{abcs}}=r_{s}i_{\text{abcs}}+L_{s}\frac{di_{\text{abcs}}}{dt}+\frac{d\Psi_{m}}{dt}$$

Defining $\omega_{r}=\frac{d\theta_{r}}{dt}$, we have:

(7) $$\frac{d\Psi_{m}}{dt}=\Psi_{m}\left[\begin{array}{c} \omega_{r}\cos(\theta_{r})\\ \omega_{r}\cos\left(\theta_{r}-\frac{2}{3}\pi \right)\\ \omega_{r}\cos\left(\theta_{r}+\frac{2}{3}\pi \right) \end{array}\right]$$

Hence, in Cauchy form, by using $L_{s}^{-1}$:

(8) $$\frac{di_{\text{abcs}}}{dt}=-L_{s}^{-1}r_{s}i_{\text{abcs}}-L_{s}^{-1}\frac{d\Psi_{m}}{dt}+L_{s}^{-1}u_{\text{abcs}}$$

Incorporating the transient behavior of the mechanical system, where electric torque *T_e_*, load torque *T_L_*, viscous friction coefficient *B_m_* and inertia moment *J* are used:

(9) $$T_{e}-B_{m}\omega_{\text{rm}}-T_{L}=J\frac{d^{2}\theta_{\text{rm}}}{dt^{2}}$$

(10) $$\frac{d\omega_{rm}}{dt}=\frac{1}{J}(T_{e}-B_{m}\omega_{rm}-T_{L})$$

where mechanical position and velocity are related by $\frac{d\theta_{rm}}{dt}=\omega_{rm}$.

To find the electromagnetic torque developed *T_e_*, where *W_PM_* is the permanent magnet energy, the co-energy *W_c_* is used:

(11) $$W_{c}=\frac{1}{2}[\begin{array}{ccc} i_{\text{as}} & i_{\text{bs}} & i_{\text{cs}} \end{array}]L_{s}\left[\begin{array}{c} i_{\text{as}}\\ i_{\text{bs}}\\ i_{\text{cs}} \end{array}\right]+[\begin{array}{ccc} i_{\text{as}} & i_{\text{bs}} & i_{\text{cs}} \end{array}]\left[\begin{array}{c} \Psi_{m}\sin(\theta_{r})\\ \Psi_{m}\sin\left(\theta_{r}-\frac{2}{3}\pi \right)\\ \Psi_{m}\sin\left(\theta_{r}-\frac{2}{3}\pi \right) \end{array}\right]+W_{PM}$$

Therefore, we have the following formula to calculate the electromagnetic torque for the three-phase *P* -pole permanent-magnet synchronous motors:

(12) $$T_{e}=\frac{P}{2}\frac{\partial \Psi_{m}}{\partial \theta_{r}}=\frac{P\Psi_{m}}{2}\left(i_{sa}\cos(\theta_{r})+i_{\text{bs}}\cos\left(\theta_{r}-\frac{2}{3}\pi \right)+i_{\text{cs}}\cos\left(\theta_{r}+\frac{2}{3}\pi \right)\right)$$

Hence:

(13) $$\frac{d\omega_{rm}}{dt}=\frac{P\Psi_{m}}{2J}\left(i_{\text{as}}\cos\left(\theta_{r}\right)+i_{\text{bs}}\cos\left(\theta_{r}-\frac{2}{3}\pi \right)+i_{\text{cs}}\cos\left(\theta_{r}+\frac{2}{3}\pi \right)\right)-\frac{B_{m}}{J}\omega_{rm}-\frac{1}{J}T_{L}$$

Using the electrical angular velocity *ω_r_* and displacement “*θ_r_*”, related to mechanical angular velocity and displacement as $\omega_{rm}=\frac{2}{P}\omega_{r}$ and $\theta_{rm}=\frac{2}{P}\theta_{r}$, results in the following equation:

(14) $$\begin{array}{c} \frac{d\omega_{r}}{dt}=\frac{P^{2}\Psi_{m}}{4J}\left(i_{\text{as}}\cos\left(\theta_{r}\right)+i_{\text{bs}}\cos\left(\theta_{r}-\frac{2}{3}\pi \right)+i_{\text{cs}}\cos\left(\theta_{r}+\frac{2}{3}\pi \right)\right)-\frac{B_{m}}{J}\omega_{r}-\frac{P}{2J}T_{L}\\ \frac{d\theta_{r}}{dt}=\omega_{r} \end{array}$$

Regarding the implicit time reparameterization to express the time functions acceleration and the speed in Equation (14) as functions of the rotor position, the relation rotor position-time is guaranteed to be invertible for the total rotor position (not only for one revolution range), if rotor position is a monotonic function of time like happen for a non-cero angular speed of constant sign.

To control the angular velocity, one regulates the currents fed or voltages applied to the stator windings. To maximize the electromagnetic torque developed, the motor should be fed by a balanced three-phase current set:

(15) $$\begin{array}{c} i_{\text{as}}\left(t\right)=\sqrt{2}i_{M}\cos\left(\omega_{r}t\right)=\sqrt{2}i_{M}\cos\left(\omega_{e}t\right)=\sqrt{2}i_{M}\cos\left(\theta_{r}\right)\\ i_{\text{bs}}\left(t\right)=\sqrt{2}i_{M}\cos\left(\omega_{r}t-\frac{2}{3}\pi \right)=\sqrt{2}i_{M}\cos\left(\omega_{e}t-\frac{2}{3}\pi \right)=\sqrt{2}i_{M}\cos\left(\theta_{r}-\frac{2}{3}\pi \right)\\ i_{\text{cs}}\left(t\right)=\sqrt{2}i_{M}\cos\left(\omega_{r}t+\frac{2}{3}\pi \right)=\sqrt{2}i_{M}\cos\left(\omega_{e}t+\frac{2}{3}\pi \right)=\sqrt{2}i_{M}\cos\left(\theta_{r}+\frac{2}{3}\pi \right) \end{array}$$

Generating an electromagnetic torque:

(16) $$T_{\text{emax}}=\frac{P\Psi_{m}}{2}\sqrt{2}i_{M}\left(\cos^{2}\left(\theta_{r}\right)+\cos^{2}\left(\theta_{r}-\frac{2}{3}\pi \right)+\cos^{2}\left(\theta_{r}+\frac{2}{3}\pi \right)\right)=\frac{3P\Psi_{m}}{2\sqrt{2}}i_{M}$$

To produce the specified current, the balanced three-phase voltages are given as:

(17) $$\begin{array}{c} u_{\text{as}}\left(t\right)=\sqrt{2}u_{M}\cos\left(\theta_{r}\right)\\ u_{\text{bs}}\left(t\right)=\sqrt{2}u_{M}\cos\left(\theta_{r}-\frac{2}{3}\pi \right)\\ u_{\text{cs}}\left(t\right)=\sqrt{2}u_{M}\cos\left(\theta_{r}+\frac{2}{3}\pi \right) \end{array}$$

To simplify the control of PMSM, it is a common practice to transform the equations from three-phase voltages ***u****_abcds_* to the *qd*0 variables ***u****_qd_*_0_*_s_* for the rotor reference frame [34].

In matrix form, the mathematical model of the PMSM in the rotor reference frame is given as:

(18) $$\left[\begin{array}{c} \frac{di_{qs}^{r}}{dt}\\ \frac{di_{ds}^{r}}{dt}\\ \begin{array}{c} \frac{di_{0s}^{r}}{dt}\\ \frac{d\omega_{r}}{dt}\\ \frac{d\theta_{r}}{dt} \end{array} \end{array}\right]=\left[\begin{array}{ccccc} -\frac{r_{s}}{L} & 0 & 0 & -\frac{\Psi_{m}}{L} & 0\\ 0 & -\frac{r_{s}}{L} & 0 & 0 & 0\\ 0 & 0 & -\frac{r_{s}}{L_{ls}} & 0 & 0\\ \frac{3P^{2}\Psi_{m}}{8J} & 0 & 0 & -\frac{B_{m}}{J} & 0\\ 0 & 0 & 0 & 1 & 0 \end{array}\right]\;\left[\begin{array}{c} i_{qs}^{r}\\ i_{ds}^{r}\\ i_{0s}^{r}\\ \omega_{r}\\ \theta_{r} \end{array}\right]+\left[\begin{array}{c} -i_{qs}^{r}\omega_{r}\\ i_{ds}^{r}\omega_{r}\\ 0\\ 0\\ 0 \end{array}\right]+\left[\begin{array}{ccc} \frac{1}{L} & 0 & 0\\ 0 & \frac{1}{L} & 0\\ 0 & 0 & \frac{1}{L_{ls}}\\ 0 & 0 & 0\\ 0 & 0 & 0 \end{array}\right]\;\left[\begin{array}{c} u_{qs}^{r}\\ u_{ds}^{r}\\ u_{0s}^{r} \end{array}\right]-\left[\begin{array}{c} 0\\ 0\\ 0\\ \frac{P}{2J}\\ 0 \end{array}\right]T_{L}$$

The required currents to regulate the angular velocity of PMSM and guarantee balanced operating conditions are given as:

(19) $$i_{qs}^{r}\left(t\right)=\sqrt{2}i_{M},i_{ds}^{r}\left(t\right)=0,i_{0s}^{r}\left(t\right)=0$$

And assuming that inductances are negligible, the applied voltages should be:

(20) $$u_{qs}^{r}\left(t\right)=\sqrt{2}u_{M},u_{ds}^{r}\left(t\right)=0,u_{0s}^{r}\left(t\right)=0$$

### 2.1. Additional Considerations

In order to understand torque ripple in PMSM we have to reconsider some assumptions from the previous model.

#### 2.1.1. Non-Sinusoidal Flux Linkages

Flux linkages are not perfectly sinusoidal so the electromotive force differs from cosine function and applying cosine currents to the stator windings produces torque ripples. The induced non-cosine electromotive forces ***e****_abcs_* are all assumed to be periodic functions with a peak value ***E****_p_* [35]:

(21) $$e_{\text{abcs}}=\frac{d\Psi_{m}}{dt}=E_{p}\omega_{r}\left[\begin{array}{c} f_{\text{as}}\left(\theta_{r}\right)\\ f_{\text{bs}}\left(\theta_{r}-\frac{2}{3}\pi \right)\\ f_{\text{cs}}\left(\theta_{r}+\frac{2}{3}\pi \right) \end{array}\right]$$

where the functions *f_as_* (*θ_r_*), *f_bs_* (*θ_r_*), and *f_cs_* (*θ_r_*), have the same shape as *e_as_, e_bs_*, and *e_cs_* with a maximum magnitude of ±1.

Since electromagnetic torque is given by:

(22) $$T_{e}=\left[e_{\text{as}}i_{\text{as}}+e_{\text{bs}}i_{\text{bs}}+e_{\text{cs}}i_{\text{cs}}\right]\frac{1}{\omega_{r}}$$

Using Equation (21), we finally have:

(23) $$T_{e}=E_{p}\left[f_{\text{as}}\left(\theta_{r}\right)i_{\text{as}}+f_{\text{bs}}\left(\theta_{r}-\frac{2}{3}\pi \right)i_{\text{bs}}+f_{\text{cs}}\left(\theta_{r}+\frac{2}{3}\pi \right)i_{\text{cs}}\right]$$

where *T_e_* is still independent of frequency, but in this case current waveforms should be calculated to produce constant torque.

#### 2.1.2. Non-Constant Inductances

From Equation (1), electromotive force can be calculated by:

(24) $$e_{\text{abcs}}=\frac{d\Psi_{\text{abcs}}}{dt}=\frac{d}{dt}[L_{s}i_{\text{abcs}}+\Psi_{m}]$$

Without assuming constant ***L****_s_*:

(25) $$e_{\text{abcs}}=L_{s}\frac{di_{\text{abcs}}}{dt}+i_{\text{abcs}}\frac{dL_{s}}{dt}+\frac{d\Psi_{m}}{dt}$$

The second term from Equation (25) produces torque ripple due to inductance angular variations, and it is associated to differences in winding inductances of the stator.

#### 2.1.3. Stator Yoke Reluctance Variations

From a macroscopic viewpoint, the torque produced in a PMSM is given by [35]:

(26) $$T_{e}=\frac{1}{2}i^{2}\frac{dL}{d\theta }-\frac{1}{2N^{2}}{\Psi_{m}}^{2}\frac{dR}{d\theta }+i\frac{d\Psi_{m}}{d\theta }$$

As mentioned before, the first term appears when motor construction causes the winding inductance to vary as a function of position, and third term describes the mutual torque that is used to make the motor shaft turn. Additionally the second term describes cogging torque that appears whenever rotor magnetic flux travels through the varying reluctance of stator yokes, attempting to align with the stator teeth or poles independent of any current. When motor shaft is rotated by hand, the pulsations felt are caused by cogging torque.

## 3. Self-Tuning Fourier Coefficient Algorithm

As stated in the previous section, torque ripples arise from non-sinusoidal flux density distribution around the air-gap and variable magnetic reluctance due to stator slots distribution. These torque ripples change periodically with rotor position and are apparent as speed variations particularly at low speeds.

These periodic torque ripples *T_r_*(*θ_r_*), with period *τ* = 2π can be represented in the form of a Fourier series:

(27) $$T_{r}\left(\theta_{r}\right)=a_{0}+\sum_{k=1}^{N}\left[a_{k}\cos\left(k\theta_{r}\right)+b_{k}\sin\left(k\theta_{r}\right)\right]$$

where *a*_0_, *a_k_*, and *b_k_* are unknown constant vectors.

Since cogging torque and harmonic components of the non-sinusoidal electromotive force depend on the slot distribution, torque ripple is a periodic function of the position and can be considered anti-symmetric and modeled by the sinusoidal components.

(28) $$T_{r}\left(\theta_{r}\right)=\sum_{k=1}^{N}\left[b_{k}\sin\left(k\theta_{r}\right)\right]$$

Considering the inertia moment of the system *I*, the acceleration *a_r_* angular velocity *ω_r_*, in relation to θ*_r_*, are given by:

(29) $$\alpha_{r}\left(\theta_{r}\right)=\frac{T_{r}\left(\theta_{r}\right)}{I}=\frac{1}{I}\left(\sum_{k=1}^{N}\left[b_{k}\sin\left({k\theta }_{r}\right)\right]\right)$$

(30) $$\omega_{r}\left(\theta_{r}\right)=\frac{1}{I}\left(\sum_{k=1}^{N}\left[-\frac{b_{k}}{k}\cos\left({k\theta }_{r}\right)\right]\right)$$

To compensate for the velocity ripple a control voltage *u*(*t*) should be introduced where *K_v_* is the voltage constant:

(31) $$u\left(\theta_{r}\right)=\frac{K_{v}}{I}\left(\sum_{k=1}^{N}\left[\frac{b_{k}}{k}\cos\left(k\theta_{r}\right)\right]\right)$$

Introducing $c_{k}=\frac{K_{v}b_{k}}{Ik}$, the control voltage can be written, as function of the position θ*_r_*(*t*), as:

(32) $$u\left(\theta_{r}\right)=\sum_{k=1}^{N}\left[c_{k}\cos\left(k\theta_{r}\left(t\right)\right)\right]$$

For each term *c_k_* cos(*k_θr(t)_*) the angular position ripple θ*_ripp_*(*t*) can be approximated by a sinusoidal function, and because of speed variations its temporal representation of cos(*k*θ*_r_*(*t*)) is distorted, thus we can use a measure of its distortion S to iteratively adjust its coefficients *c_k_* until distortion is reduced by regulating *ω_r_*(*_θr_*). Let τ =2π be the spatial period for a complete mechanical revolution:

(33) $$S=\int_{0}^{\tau }\cos\left(k\theta_{\text{ripp}}\left(t\right)\right)dt$$

(34) $$c_{k}\left(t\right)=c_{k}\left(t-1\right)+\delta S$$

This algorithm permits adjusting the control voltage parameters, adapting for changes in the torque ripple, and the parameter δ allows for controlling the adjusting speed.

### Demonstration

While the shape of the cogging torque is a complex function of motor geometry and material properties, here it is approximated by a sinusoidal function and consequently the angular position ripple is also approximated by a sinusoidal function.

Assuming that angular position is given by *θ_r_(t)* = *ω*_0_*t* + *Asin* (*ω*_0_*t* + *φ*), were nominal angular position “*ω*_0_*t*” comes from the nominal constant speed of the rotor “*ω*_0_”, and the angular position ripple “*Asin*(*ω*_0_*t* + *φ*)” corresponds to the angular lag or advance (from nominal angular position), produced by periodic perturbation such as cogging torque. Then the angular velocity *ω_r_*, and angular position ripple θ*_ripp_* are given by:

(35) $$\theta_{\text{ripp}}\left(t\right)=\text{as}in\left(\omega_{0}t+\varphi \right)$$

(36) $$\omega_{r}\left(t\right)=\omega_{0}+A\omega_{0}\cos\left(\omega_{0}t+\varphi \right)$$

For each term *c_k_* cos(*k*θ*_r_*(*t*)), according to Fourier series methodology, its value is in theory determined by:

(37) $$c_{k}=\frac{1}{T}\int_{0}^{T}\cos\left({k\theta }_{\text{ripp}}\left(t\right)\right)\omega_{r}\left(t\right)dt$$

were T represent the temporal period for a complete mechanical revolution.

In practice, *ω_r_*(*t*) is not necessarily a periodic function of time and synchronization of the controller to the torque perturbation could be difficult to achieve.

Using Equations (35) and (36) in Equation (37):

(38) $$c_{k}=\frac{1}{T}\int_{0}^{T}\cos\left(\text{k Asin}\left(\omega_{0}t+\varphi \right)\right)\left(\omega_{0}+{A\omega }_{0}\cos\left(\omega_{0}t+\varphi \right)\right)dt$$

So:

(39) $$c_{k}=\frac{\omega_{0}}{T}\int_{0}^{T}\cos\left(k\text{as}in\left(\omega_{0}t+\varphi \right)\right)dt+\frac{1}{T}\int_{0}^{T}A\omega_{0}\cos\left(\omega_{0}t+\varphi \right)\cos\left(k\text{as}in\left(\omega_{0}t+\varphi \right)\right)dt$$

Recognizing that the second term is equal to zero, because can be rewritten as $\frac{1}{Tk}\int_{0}^{T}\cos\left(u\right)du$:

(40) $$c_{k}=\frac{\omega_{0}}{T}\int_{0}^{T}\cos\left(\text{kAsin}\left(\omega_{0}t+\varphi \right)\right)dt$$

Finally:

(41) $$c_{k}=\frac{\omega_{0}}{T}\int_{0}^{T}\cos\left({k\theta }_{\text{ripp}}\left(t\right)\right)dt$$

when the rotor speed is not constant or periodic of unknown period it is preferable to replace T by τ to allow for a fix parameter. Furthermore instead of computing $\frac{\omega_{0}}{T}$, because the angular velocity is changing, the integral term can be used to iteratively adjust the coefficient *c*, through the gain factor δ as proposed in Equations (33) and (34).

## 4. Experimental Implementation

**Figure 1.**
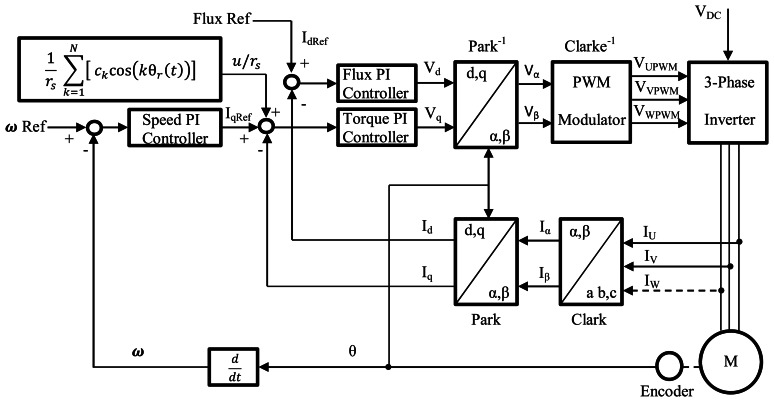
Block diagram of the Fourier series controller applied to the Field Oriented Control.

Figure 1 shows the overall torque ripple minimization scheme. During the transient state, the Fourier Series Controller is not activated and the Field Oriented Control (FOC) sets the motor in a stable operation.

When steady state is reached, the Fourier Series Controller is applied and it provides the additional compensation so as to minimize torque ripple. Conventional PI current controllers that generate the control voltages in accordance with the field oriented control are used in the inner loop. The current controllers work with a sample time of 500 μs and gains are set as: K_p_ = 1, K_i_ = 80, all variables are considered in per unit values and the δ parameter is set to 0.02.

**Figure 2.**
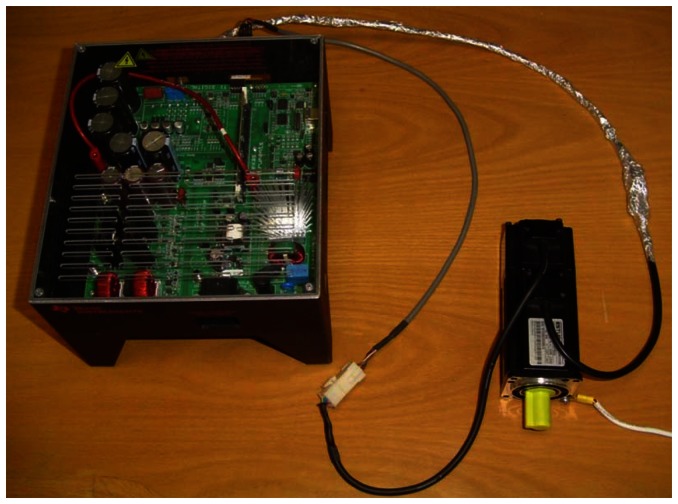
Experimental setup including the TMDSHVMTRPF development and EMJ-04APB22 PMSM.

Figure 2 shows the configuration of the experiments. A TMDSHVMTRPF development system with a F28035 DSP control card is connected to the EMJ-04APB22 four pole pairs Permanent Magnet Synchronous Motor with the following parameters: 200 V, 2.7 A, 400 W maximum power, 300 rpm rated speed, 4.7 Ω, 0.014 H stator resistance and inductance, and 2500 PPR incremental encoder attached. Measurements of the variables were taken at the PWMDAC ports of the TMDSHVMTRPF development system by using a FLUKE 199C floated oscilloscope-meter, setting the bandwidth to 10 kHz for high frequency rejection. The encoder signals were coupled with a TTL Buffer (SN74LS243N Bus Transceiver), to avoid electric noise that degrades the angular position signal readings.

The performance evaluation of the controller with the proposed self-tuning algorithm is presented in the following section.

## 5. Experimental Results

To verify the performance of the proposed self-tuning algorithm with the Fourier series expansion scheme, experiments were performed using the setup described in the preceding section. The experiments were conducted for speeds lower than 10% of the motor's nominal speed. The performance criterion used to evaluate the performance of the proposed scheme for torque ripple minimization is the variation of the angular speed determined from the angular position measurements from the encoder.

### The Standard Field Oriented Control

**Figure 3.**
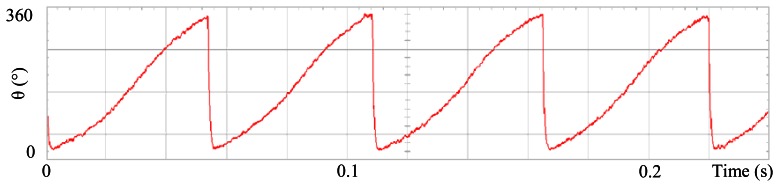
Angular position of the motor, at 273 rpm, controlled by Field Oriented Control.

**Figure 4.**
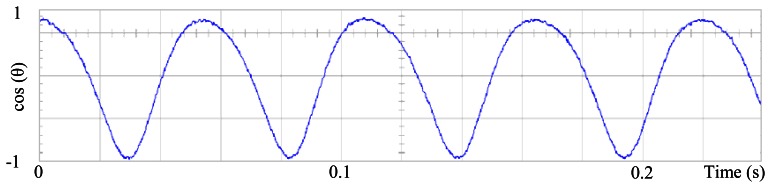
Fundamental cosine term, distorted by speed ripple, for Field Oriented Control.

**Figure 5.**
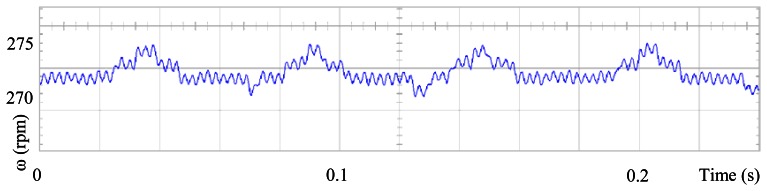
Speed ripple, at 273 rpm, controlled by Field Oriented Control.

Figure 3 shows the angular position of the motor versus time, the slope of the triangular waveform change reflecting speed variations. In Figure 4 a cosine function of the angular position, with the same frequency as the perturbation fundamental frequency, is plotted against time, showing a distortion shape caused by speed variation that can be used to adjust the Fourier coefficients. Figure 5 presents the speed ripple, although it is only 1.6% of the reference speed (4.4 rpm of 273 rpm), it is responsible for distorting the previous two waveforms.

**Figure 6.**
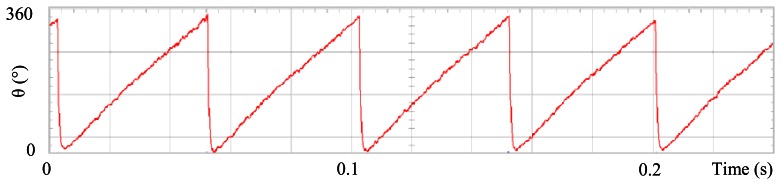
Angular position of the motor, at 273 rpm, controlled by the proposed scheme, for the first two terms.

**Figure 7.**
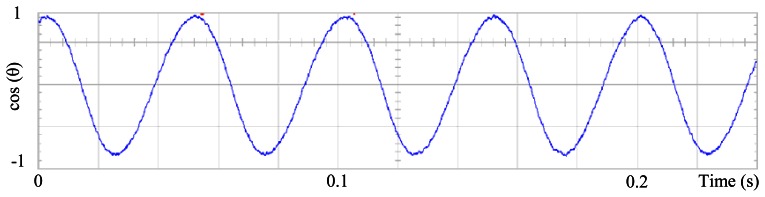
Fundamental cosine term, corrected by the proposed scheme, for the first two terms.

**Figure 8.**
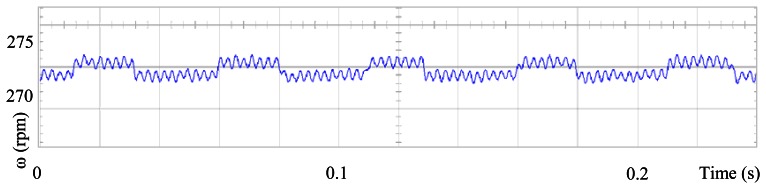
Speed ripple, at 273 rpm, controlled by the proposed scheme, for the first two terms.

**Figure 9.**
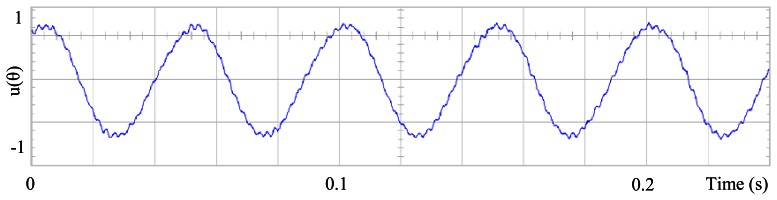
Control signal u(θ), of the proposed scheme, for the first two terms.

The proposed self-tuning algorithm with the Fourier series expansion scheme (for the first two terms) Figure 6 shows a reduction in angular position waveform distortion. Figure 7 verifies that the fundamental cosine function shape also improved. Figure 8 presents a 2 rpm peak to peak speed ripple which is half of the original variation as shown in Figure 5. The waveform in Figure 9 corresponds to the control signal u(θ) in per unit values.

**Figure 10.**
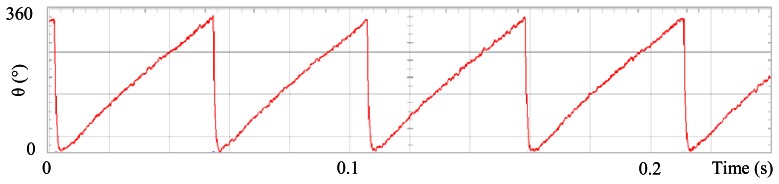
Angular position of the motor, at 273 rpm, controlled by the proposed scheme, for the first four terms.

**Figure 11.**
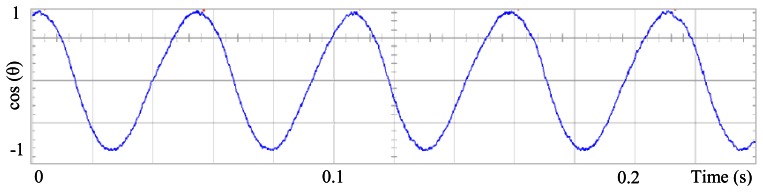
Fundamental cosine term, corrected by the proposed scheme, for the first four terms.

**Figure 12.**
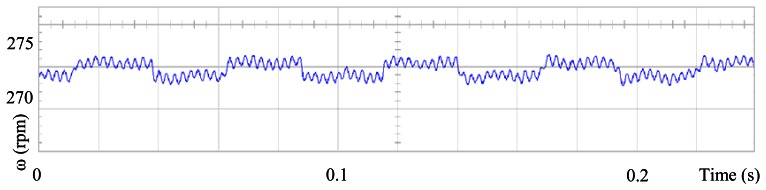
Speed ripple, at 273 rpm, controlled by the proposed scheme, for the first four terms.

**Figure 13.**
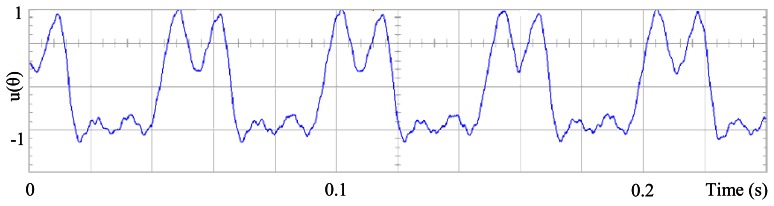
Control signal u(θ), of the proposed scheme, for the first four terms.

**Figure 14.**
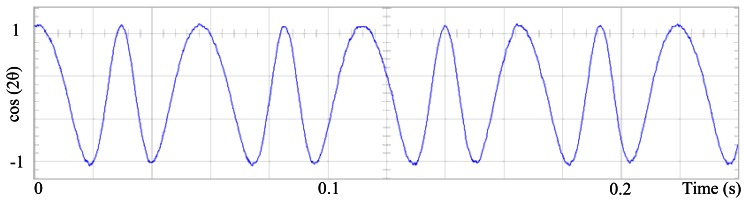
Second harmonic cosine term, distorted by speed ripple, for Field Oriented Control.

**Figure 15.**
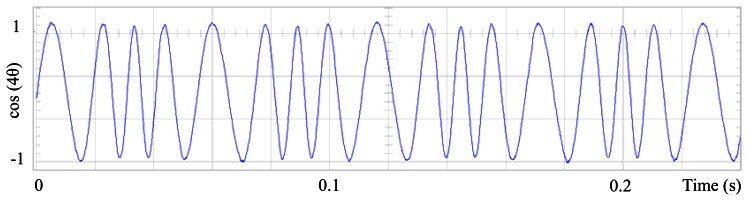
Fourth harmonic consine term, distorted by speed ripple, for Field Oriented Control.

**Figure 16.**
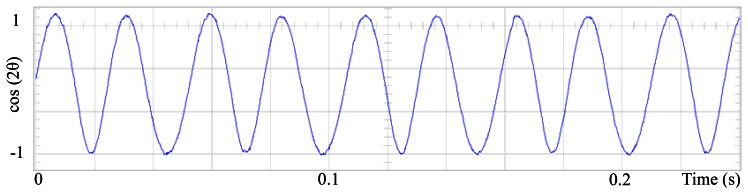
Second harmonic cosine term, corrected by the proposed schem.

**Figure 17.**
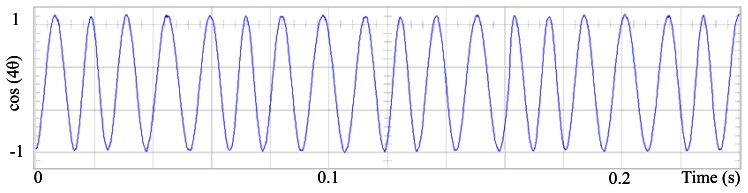
Fourth harmonic consine term, corrected by the proposed scheme.

**Figure 18.**
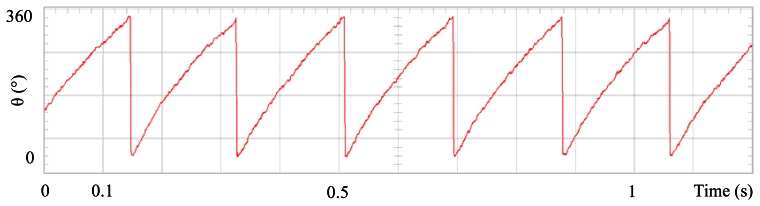
Angular position of the motor, at 80 rpm, controlled by the proposed scheme.

Figures 10, 11 and 12 shows similar results for the proposed control for the first four terms. Figure 13 presents the control signal u(θ), although the wave forms in Figures 9 and 13 are substantially different, for this experiment the results are similar and perhaps it is because torque ripple is almost sinusoidal. Figures 14, 15, 16 and 17 shows that the second and fourth harmonic cosine function shape also improved. Finally Figure 18 shows that speed can be reduced to 2.7% of nominal speed while the motor starts to malfunction at 5.7% of nominal speed with only FOC control.

## 6. Conclusions

In this paper has been presented an adaptive self-tuning algorithm for determining the Fourier coefficients of the controller with the aim of reducing the torque ripple in a PMSM. Its implementation is simple and represents a good alternative for minimizing torque ripple, cogging torque and non-sinusoidal electromotive torque variations due to its periodic nature. The proposed scheme does not require previous knowledge of the motor parameters. The performance of the proposed scheme has been evaluated through experimentation and test results confirm 50% speed ripple reduction (from 4.4 rpm to 2 rpm peak to peak speed ripple). Further research should be conducted to extend these results to applications where load torque varies as periodic function, which is not considered in this work.
